# New Conformational State of NHERF1-CXCR2 Signaling Complex Captured by Crystal Lattice Trapping

**DOI:** 10.1371/journal.pone.0081904

**Published:** 2013-12-10

**Authors:** Yuanyuan Jiang, Guorong Lu, Laura R. Trescott, Yuning Hou, Xiaoqing Guan, Shuo Wang, Angelique Stamenkovich, Joseph Brunzelle, Nualpun Sirinupong, Chunying Li, Zhe Yang

**Affiliations:** 1 Department of Biochemistry and Molecular Biology, Wayne State University School of Medicine, Detroit, Michigan, United States of America; 2 Advance Photon Source, Argonne National Lab, Argonne, Illinois, United States of America; 3 Nutraceuticals and Functional Food Research and Development Center, Prince of Songkla University, Hat-Yai, Songkhla, Thailand; University of Oulu, Finland

## Abstract

NHERF1 is a PDZ adaptor protein that scaffolds the assembly of diverse signaling complexes and has been implicated in many cancers. However, little is known about the mechanism responsible for its scaffolding promiscuity or its ability to bind to multiple targets. Computational studies have indicated that PDZ promiscuity may be attributed to its conformational dynamics, but experimental evidence for this relationship remains very limited. Here we examine the conformational flexibility of the NHERF1 PDZ1 domain using crystal lattice trapping via solving PDZ1 structure of a new crystal form. The structure, together with prior PDZ1 structures of a different space group, reveals that 4 of 11 ligand-interacting residues undergo significant crystal packing-induced structural changes. Most of these residues correspond to the residues involved in allosteric transition when a peptide ligand binds. In addition, a subtle difference in ligand conformations causes the same peptide to bind in slightly different modes in different crystal forms. These findings indicate that substantial structural flexibility is present in the PDZ1 peptide-binding pocket, and the structural substate trapped in the present crystal form can be utilized to represent the conformational space accessible to the protein. Such knowledge will be critical for drug design against the NHERF1 PDZ1 domain, highlighting the continued need for experimentally determined PDZ1-ligand complexes.

## Introduction

The Na^+^/H^+^ exchanger regulatory factor 1 (NHERF1) is a multifunctional scaffold protein that plays a central role in diverse cellular events through recruiting receptors, transporters, and signaling molecules into specific functional complexes[1]. NHERF1 also plays a significant role in multiple cancers where its elevated expression correlates with aggressive stage and poor overall prognosis[2]. The functional diversity of NHERF1 in normal and pathological conditions depends largely on its two PDZ (PSD-95/Discs-large/ZO-1) domains, PDZ1 and PDZ2, which are highly promiscuous and capable of interacting with a large number of biologically different proteins[1]. To date, over 40 binding partners of NHERF1 have been identified; most of which are membrane receptors and transporters, such as the interleukin 8 receptor beta (CXCR2), the cystic fibrosis transmembrane conductance regulator (CFTR), the β2-adrenergic receptor (β2AR), the platelet-derived growth factor receptor (PDGFR), and the parathyroid hormone receptor (PTHR)[3], [4], [5], [6], [7]. Through these PDZ-interacting proteins, NHERF1 regulates many processes, including cell proliferation, invasion and migration, signal transduction, and protein trafficking[8], [9]. Our recent studies showed that the PDZ domains of NHERF1 bind CXCR2 in neutrophils, regulating neutrophil chemotaxis and directing neutrophils to sites of inflammation[3]. A similar interaction was observed for pancreatic cancers, where disruption of this PDZ-mediated interaction was capable of suppressing human pancreatic tumor growth in vivo[7]. These recent evidence suggests targeting the PDZ-mediated NHERF1-CXCR2 interaction may represent a novel clinical strategy, which could be valuable in the development of new treatments against numerous neutrophil-dependent inflammatory diseases as well as pancreatic cancers[8], [9].

In general, PDZ domains mediate protein interactions by recognizing the C-terminal sequence of target proteins, and by binding to the targets through a canonically and structurally-conserved PDZ peptide binding pocket[10]. Based on the residues at positions 0 and -2 of the peptides (position 0 referring to the C-terminal residue), early studies grouped PDZ domains into two major specificity classes: class I, (S/T)X(V/I/L) (X denoting any amino acid); class II, (F/Y)X(F/V/A) [11], [12], [13]. However, growing evidence indicates that PDZ specificity is unexpectedly complex and diverse, with the PDZ domain family recognizing up to 7 C-terminal ligand residues and forming at least 16 unique specificity classes[14]. In addition, the complexity of PDZ-peptide interactions is exemplified by the facts that many PDZ domains can bind to multiple ligands of different peptide classes, and that single peptides are able to bind to distinct PDZ domains[14]. This complex picture of PDZ-peptide interactions raises a challenging problem regarding how PDZ domains, structurally simple protein interaction modules, achieve binding promiscuity and specificity concomitantly, the nature of which remains obscure.

Because promiscuity and specificity have important implications in highly selective drug design[15], understanding the mechanism that determines PDZ interaction with specific peptide sequences is a subject of intensive research. Recent binding specificity studies of 157 mouse PDZ domains revealed that PDZ domains are evenly distributed throughout selectivity space, suggesting that they have been optimized across the proteome to minimize cross-reactivity[16]. The same study revealed a weak but significant correlation between the pairwise sequence divergence of PDZ domains and their divergence in ligand selectivities[16]. More recent specificity profiling studies with 91 point mutants of a model PDZ domain revealed that PDZ binding preference can be influenced by multiple structural and chemical mechanisms involving both direct contacts and cooperative, long-range effects, suggesting that PDZ specificity evolves rapidly, thus enabling PDZ for robust interaction with many biologically distinct ligands[14]. Using shotgun alanine scanning, another PDZ specificity study has yielded considerable insights into the relationships between primary sequence and specificity[17]. This study demonstrated that most of the alanine substitutions in HtrA1-PDZ are neutral with respect to peptide-binding selectivity and only a subset of mutations, mostly within the canonical PDZ binding pocket, affects its binding specificity[17]. Therefore, the results of these studies have offered considerable information about how the sequence composition determines PDZ specificity, and a coherent picture of their relationships is beginning to emerge.

Despite the wealth of detail about PDZ specificity, the mechanism that determines PDZ promiscuity still remains poorly understood, partly because it has been difficult to explain PDZ promiscuity simply based on its sequence composition. It is important to note that a number of computational and experimental studies have suggested the conformational dynamics of PDZ domains may play a crucial role in ligand binding, especially in determining binding promiscuity[18], [19], [20], [21]. For example, molecular dynamics simulation of 12 PDZ domains revealed that binding dynamics and entropy are extremely variable not only across PDZ domains but also for the same PDZ domain bound with different ligands[18]. This indicates that complex-specific dynamical or entropic responses may form the basis for promiscuous binding and sustaining promiscuity in highly selective PDZ-peptide interactions[18]. Another computational study of five different PDZ domains came to similar conclusion. It revealed the existence of a close relationship between intrinsic dynamics and binding promiscuity and suggested the ability of PDZ domains to interact with multiple ligands requires the binding pocket to adopt significantly different conformations[19]. In addition, based on differential domain fluctuation profiles, the latter study also indicated that both induced fit and conformational selection play roles in PDZ ligand binding, but the extent to which these mechanisms are involved is highly variable across the PDZ domain family[19]. Remarkably, recent NMR dynamics studies demonstrated that the ligand-bound conformation is already present in the conformational ensemble populated by unliganded protein, suggesting the intrinsicality of protein to fluctuate between multiple conformers, or conformational selection, might be the fundamental paradigm for promiscuous ligand binding[22], [23], [24]. These studies made it apparent that detailed and comparative analysis of PDZ conformational plasticity will be required to establish and illuminate the full range of ligand promiscuity specified by the PDZ domain fold. A high-resolution structural interpretation of individual conformational states should in turn provide considerable insights into the mechanisms whereby the exquisite ligand promiscuity dictates the diversification of biological functions.

In order to understand the promiscuity and specificity of the NHERF1 PDZ domains, we have previously reported a high-resolution PDZ1 crystal structure in complex with the CXCR2 C-terminal sequences[6]. We found that NHERF1 PDZ1 is capable of assuming distinct conformational states when the structure of PDZ1-CXCR2 was compared to the structures of three other PDZ1 complexes, including PDZ1-CFTR, PDZ1-PDGFR, and PDZ1-β2AR[13], [25]. Importantly, the complex-specific conformations were found to be closely associated with the various characteristics of peptide ligands[6], [25], suggesting that PDZ1 promiscuity is facilitated by protein flexibility that allows robust accommodation of peptides with distinct sequences. While these studies provided valuable insight about PDZ1 promiscuity and flexibility, the questions still remain concerning the dynamical features that control explicit binding of each of PDZ1 ligands and whether NHERF1 function relies on PDZ1 conformational diversity. Additionally, we are still far from a complete description of PDZ1 conformational space, and the amount of available PDZ1 structures may represent only a tiny fraction of the entire ensemble[18], [19], [20], [21]. It is conceivable that limited numbers of PDZ1 structures could limit their usefulness in rational drug design owing to large unexplored conformational space that may compensate drug discovery efforts for potency and selectivity. Moreover, the lack of a complete picture of PDZ1 conformational space could lead to an incomplete understanding of the complex relationship between PDZ1 conformational dynamics and the promiscuous nature of its substrate specificity. In these regards, we here present a new conformational state of PDZ1 by solving the structure of the PDZ1-CXCR2 complex in a new crystal form. Multiple PDZ1 conformations observed in the present crystal form and another crystal form reported previously[6] provide an additional insight into PDZ1 conformational dynamics and a structural explanation for how PDZ1 is able to bind to different ligands. Alternatively, the variations in the structures of different crystal forms raise the challenge for selective drug design, emphasizing the need for obtaining X-ray crystal structures of various PDZ1 conformational states to inform the drug design process.

## Results

### New Crystal Form of PDZ1-CXCR2 Complex

Alternative crystal forms can trap a protein in different conformational states, providing snapshots of the conformations accessible to the protein[26], [27], [28]. To reveal possible PDZ1 conformational states and how these may be important for PDZ1 promiscuity, we sought to use this well-recognized strategy to improve our understanding on PDZ1 conformational dynamics. Previously, we crystallized the PDZ1-CXCR2 complex in the *P*3_1_21 space group and have determined its structure at 1.16 Å resolution (*P*3_1_21-PDZ1)[6]. In the current study, by using different crystallizing precipitant under similar pH, we obtained a new crystal form that diffracted to 1.10 Å resolution. The new crystal belongs to the *P*2_1_ space group (*P*2_1_-PDZ1), and the structure was solved by molecular replacement. The model was refined to R_work_ of 14.3% and R_free_ of 15.6%, and the validation of its stereochemistry using Molprobity[29] showed that 97.9% of the residues are in the most favored regions, 2.1% in the additional allowed regions, and 0.0% in the disallowed regions (Table 1).

**Table 1:** Crystallographic data and refinement statistics.

Space group	*P*2_1_	*P*3_1_21
Cell parameters (Å)		
a	26.6	50.4
b	45.5	50.4
c	33.4	66.0
Wavelength (Å)	0.97856	0.97872
Resolution (Å)	45.5−1.1 (1.16−1.10)^a^	20.0−1.16 (1.20−1.16)
*R_merge_* b	0.024 (0.180)	0.063 (0.463)
*R*_meas_ c	0.034 (0.248)	0.068 (0.543)
CC_1/2_ d	0.999 (0.951)	0.999 (0.924)
Redundancy	3.7 (2.4)	9.7 (7.0)
Unique reflections	30032	33912
Completeness (%)	97.7 (86.0)	100 (100)
〈I/σ〉	24.8 (4.5)	19.1 (3.3)
Wilson B-factor	8.2	10.1
**Refinement**		
Resolution (Å)	25.9−1.10	20.0−1.16
Molecules/AU	1	1
*R_work_* e	0.143 (0.158)	0.148 (0.209)
*R_free_* f	0.156 (0.193)	0.163 (0.236)
Ramachandran plot by Molprobity		
Residues in favored region	97.9%	97.9%
Residues in allowed region	2.1%	2.1%
RMSD		
Bond lengths (Å)	0.010	0.007
Bond angels (°)	1.2	1.2
No. of atoms		
Protein (residues 9–94)	679	679
Peptide (residues 95–99)	39	39
Water	161	105
Chloride	1	4
Acetate	4	
B-factor (Å^2^)		
Protein	14.8	17.2
Peptide	9.7	13.7
Water	26.3	25.7
Chloride	16.2	16.4
Acetate	23.4	

a Numbers in parentheses refer to the highest resolution shell.

b *R_merge_* = Σ|I−〈I〉|/ΣI, where I is the observed intensity and 〈I〉 is the averaged intensity of multiple observations of symmetry-related reflections.

c *R_meas_* = Σ[(n/n−1)]^1/2^Σ|I−〈I〉|/ΣI, where n is the number of observations of a given reflection.

d Half-dataset correlation coefficient.

e *R_work_* = Σ|F_o_−F_c_|/Σ|F_o_|, where F_o_ is the observed structure factor, F_c_ is the calculated structure factor.

f *R_free_* was calculated using a subset (5%) of the reflection not used in the refinement.

**Figure 1:**
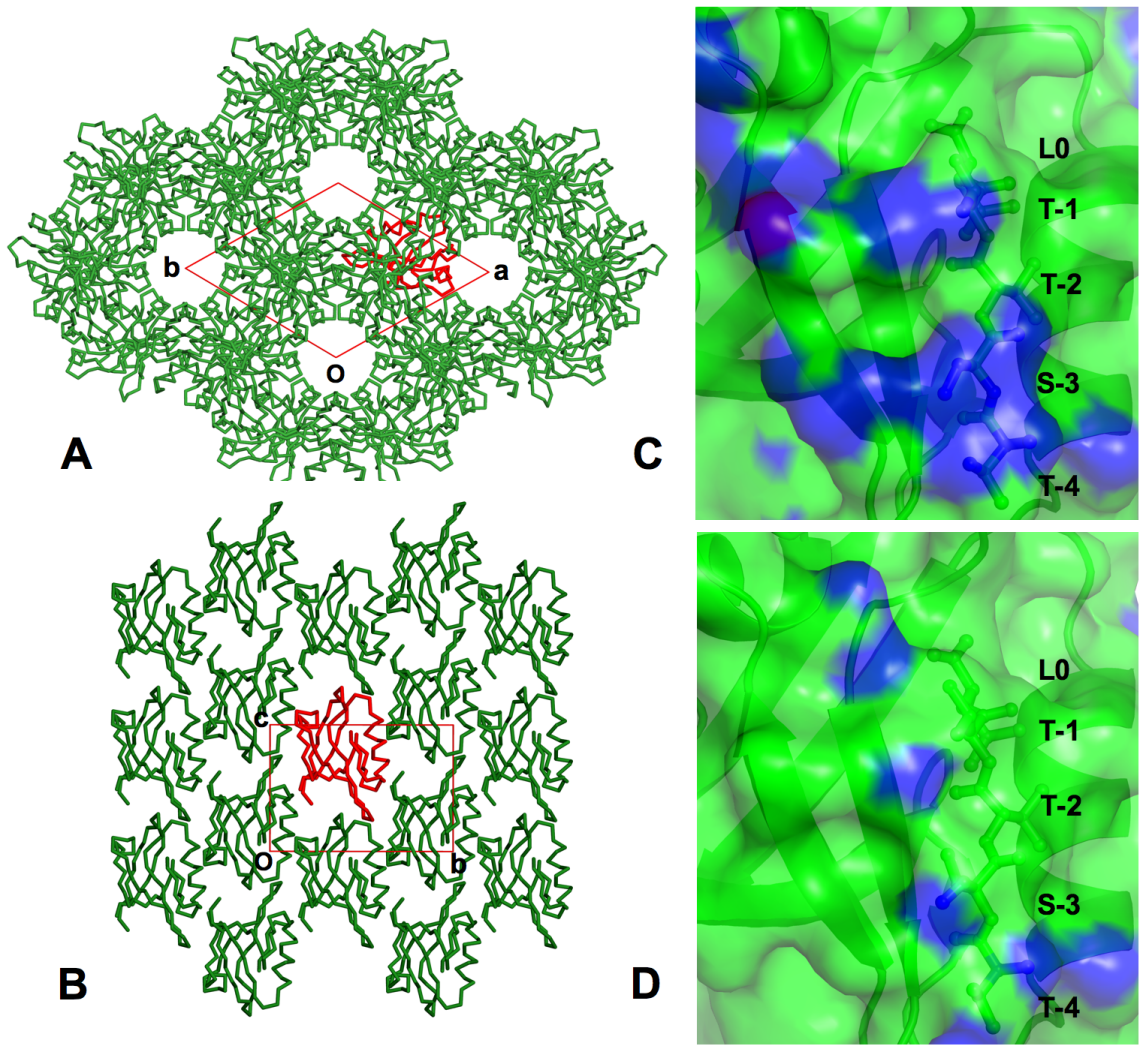
Crystal packing differences between two crystal forms. (A) Section of the crystal lattice of *P*3_1_21-PDZ1 and (B) *P*2_1_-PDZ1. The unit cell is shown as a red box, with the origin and axes labeled. PDZ1 is shown as a Cα trace, with red standing for a reference molecule and green the symmetry-related molecules. (C) Surface representation of crystal contacts around the ligand-binding site of *P*3_1_21-PDZ1 and (D) *P*2_1_-PDZ1. The surface is colored in blue if the distance to symmetry-related molecules is 3.5 Å or less and is colored in green otherwise. PDZ1 is depicted as ribbon and the bound CXCR2 peptide is labeled and represented by sticks.

Both crystal forms contain one molecule per asymmetric unit, but their crystal packing environments differ significantly. For *P*3_1_21 the distinctive packing pattern is manifested by linear stacking of PDZ1 complexes, hexagonal lateral association, and the existence of large solvent channels across the crystal (Figure 1A). In the case of *P*2_1_, the PDZ1 complexes are stacked in a staggered arrangement, displaying a densely packed, flattened configuration (Figure 1B). Consistent with the packing environments, the solvent content in the *P*3_1_21 crystal form is higher than *P*2_1_-PDZ1, ∼50% compared to ∼37%. However, analysis of crystal contacts reveals that there are more intimate packing interactions in the *P*3_1_21 crystal. For example, with distances of less than 3.5 Å defined as contacts, *P*3_1_21 has 128 crystal contacts with symmetry-related molecules, whereas the *P*2_1_ crystal has only 82 such contacts. Accessibility calculation with AREAIMOL[30], [31] shows that 2608 Å^2^ of protein surface is buried by symmetry-related molecules in *P*3_1_21-PDZ1, compared to only 2382 Å^2^ buried in *P*2_1_-PDZ1. Thus, it appears that the protein molecules in the *P*2_1_ crystal pack more loosely than in *P*3_1_21-PDZ1, though it has a relatively lower solvent content. Furthermore, their distinct packing environments are highlighted by strikingly large differences in their crystal contact surfaces. For all of the 128 contacts sites found in *P*3_1_21, there is no corresponding contact surface with equivalent residue composition in *P*2_1_-PDZ1 (Figure 1C and 1D). This difference provides the basis for us to utilize crystal packing in understanding PDZ1 conformational dynamics and should allow the capture of different conformational substates.

### Distinct PDZ1 Conformational States

**Figure 2:**
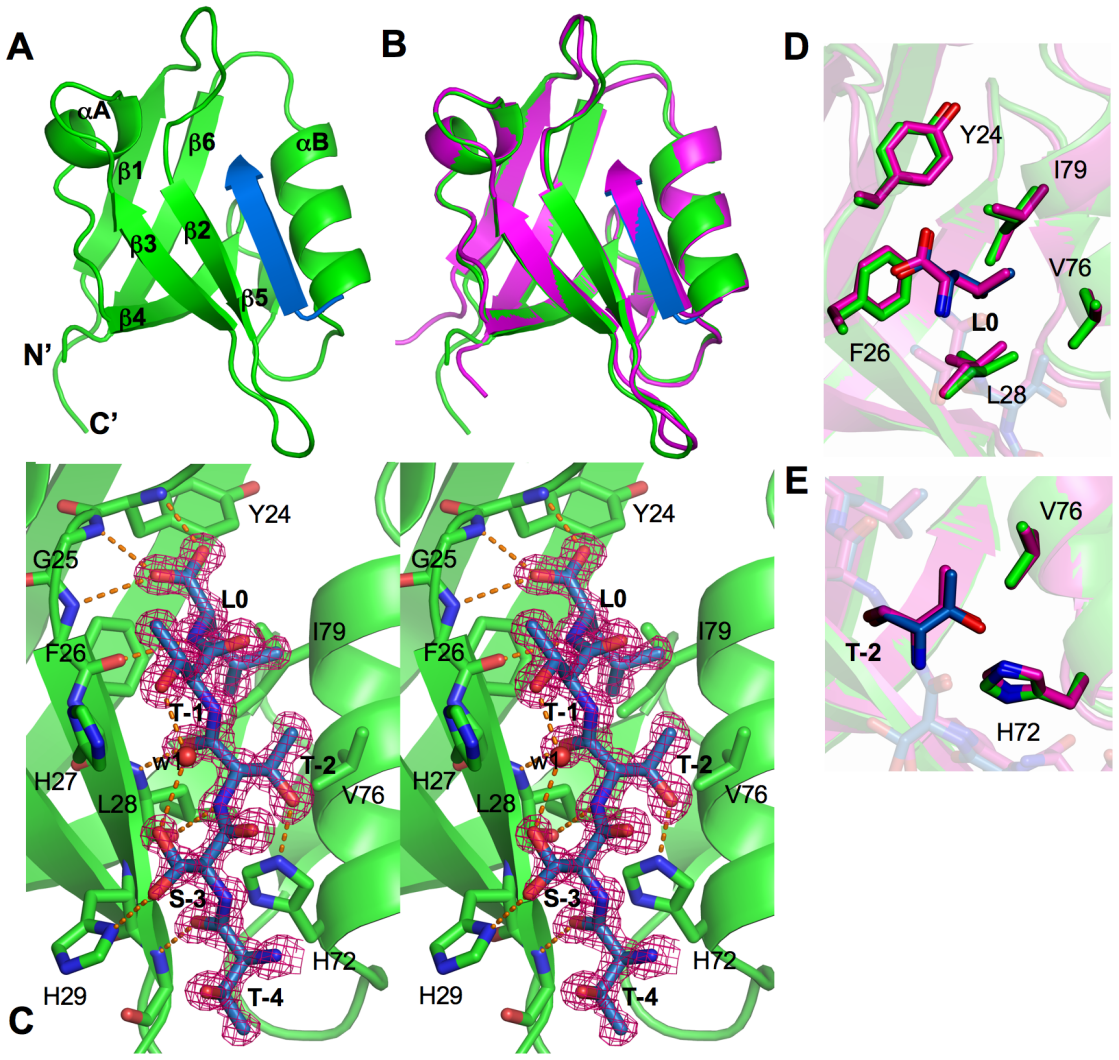
Structural similarities of two crystal forms. (A) Ribbon view of overall *P*2_1_-PDZ1 structure. PDZ1 is shown in green and the CXCR2 peptide shown in blue. Secondary structures of PDZ1, α-helices and β-strands, are labeled and numbered according to their position in the sequence. (B) Superposition of *P*2_1_-PDZ1 (green) and *P*3_1_21-PDZ1 (magenta). (C) Stereo view of the PDZ1-CXCR2 interaction in *P*2_1_-PDZ1. The PDZ1 residues are represented by sticks with their carbon atoms colored in green. The CXCR2 peptide is depicted by sticks overlaid with 2F_o_−F_c_ omit map calculated at 1.1 Å and contoured at 1.5 σ. Hydrogen bonds are illustrated as orange broken lines. (D) Superposition of the Leu0 and (E) Thr-1 recognition regions. Both *P*2_1_-PDZ1 and *P*3_1_21-PDZ1 are depicted by sticks and colored according to the scheme in Figure 2B and 2C.

Different crystal packing observed in *P*2_1_-PDZ1 and *P*3_1_21-PDZ1 indeed causes significant differences in the ligand-binding pocket, but does not alter the overall fold of the protein (Figure 2). In both crystal forms, PDZ1 adopts a conserved fold characterized by six β strands (β1–β6) and two α-helices (αA and αB). Superposition of the two structures reveals a high degree of overall structural similarity, with the rms (root-mean-square) differences of 0.91 Å for main chains and 1.46 Å for side chains. In addition, the crystal packing has little effect on the overall ligand interaction mode, as in both cases the CXCR2 peptide inserts between β2 and αB as an extra β-strand and the main-chain rms difference between the bound peptides is only 0.17 Å (Figure 2B). Moreover, closer inspection of the PDZ1-CXCR2 interactions reveals that the specific ligand recognition modes at the peptide positions 0 and -2 are virtually indistinguishable. In both crystal forms, the side chain of Leu0 is nestled in a deep hydrophobic pocket formed by structurally identical residues, including Tyr24, Phe26, and Leu28 from β2, and Val76 and Ile79 from αB (Figure 2D). At the ligand position -2, the side chain hydroxyl of Thr-2 in each structure hydrogen bonds to the imidazole ring of His72, with the side chain aliphatic carbon making contact to the structurally conserved residue Val76 (Figure 2E). It should be noted that all these CXCR2 interacting residues are spared from the crystal packing in both crystal forms, consistent with their spatially buried natures in the PDZ1-peptide complexes[6].

**Figure 3:**
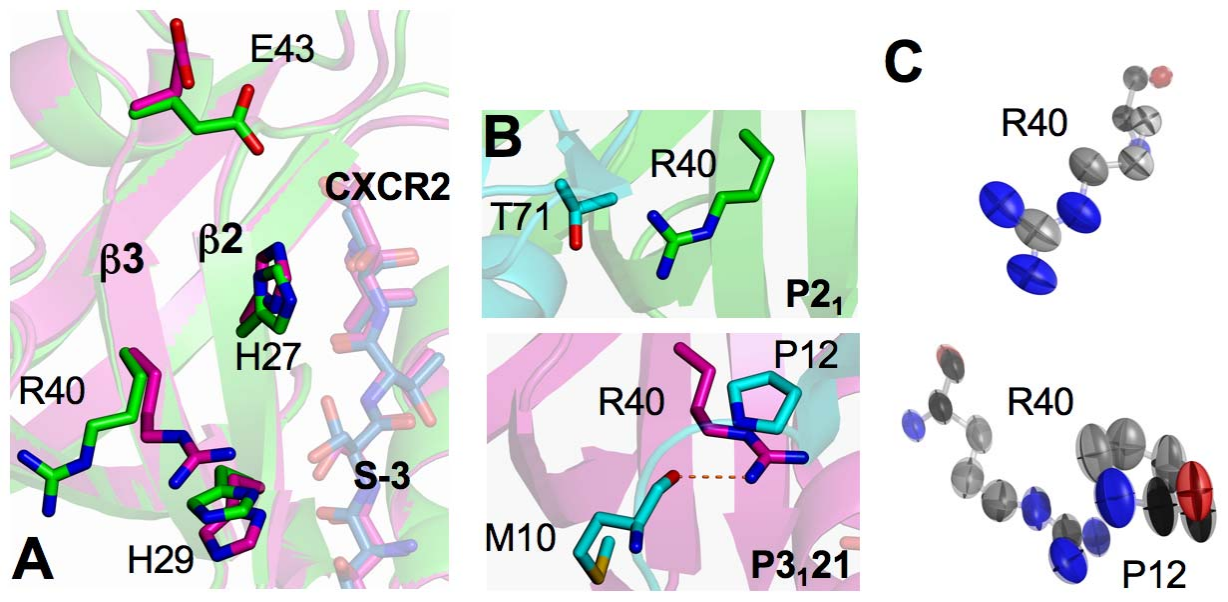
Different Arg40 conformations of two crystal forms. (A) Overall view of conformational differences in the peptide-binding pocket. *P*2_1_-PDZ1 and *P*3_1_21-PDZ1 are superimposed and colored according to the scheme in Figure 2. (B) Arg40 crystal contacts in *P*2_1_-PDZ1 (top) and in *P*3_1_21-PDZ1 (bottom). Symmetry-related molecules are represented by ribbons and sticks with their carbon atoms colored in cyan. (C) Thermal ellipsoid representation of Arg40 of *P*2_1_-PDZ1 (top) and *P*3_1_21-PDZ1 (bottom). Carbon atoms are colored gray, nitrogen atoms blue, and oxygen atoms red. Thermal ellipsoids are contoured at the 50% probability level.

In contrast to the ligand recognition at positions 0 and -2, distinct conformations between the two forms of PDZ1 structures are observed in regions that are responsible for interactions with -1 and -3 residues. Notably, the residues at these two ligand positions are highly variable across natural PDZ1 binding targets, exemplifying its ability to bind multiple targets[6]. Thus, understanding the conformational dynamics that governs the specific interactions with residues -1 and -3 should be key to understanding the underlying mechanisms of PDZ1 promiscuity. Specifically, the PDZ1 residues exhibiting large conformational differences between the two crystal forms include His27 and His29 from β2, Arg40 from β3, and Glu43 from the loop following β3 (Figure 3A). These residues are known to be important for -1 and -3 residue recognition, three of which (His29, Arg40, and Glu43) have been shown to undergo a large conformational change upon binding to different ligands[6], [25]. Remarkably, their differences in the conformations appear to be well correlated with differential crystal packing lattices, highlighting their adaptability to different environments that may be essential for PDZ1 promiscuity. In particular, the greatest difference between the ligand binding sites of the two PDZ1 forms is at residue Arg40, where the rms deviation of the main chain atoms is 0.02 Å and, for side chain atoms, 0.59 Å (Figure 3B and S1). This large variation in Arg40 conformations is closely associated with the large differences in its crystal packing environments. In *P*3_1_21-PDZ1, the side chain atoms of Arg40 make a hydrogen bond to Met10 and van der Waals contacts with the Pro12 side chain of a neighboring molecule. In *P*2_1_-PDZ1, only the Nη2 atom of Arg40 is within 4 Å distance to a neighboring Thr71, no any intermolecular contacts were observed below 3.5 Å. As a result of different crystal packing, Arg40 has very different rotameric conformations in the two crystal structures. In *P*3_1_21-PDZ1, the side chain of Arg40 is oriented toward the hydroxyl group of Ser-3, whereas in *P*2_1_-PDZ1, its guanidinium points away from the bound ligand, adopting a conformation corresponding to ∼90° rotation around the Cβ-Cγ bond of the side chain (Figure 3B). In both crystal forms, Arg40 does not make contact with the CXCR2 peptide, whereas in other PDZ1 structures bound with different peptides[13], [25], it is a key anchor residue for specific Asp-3 recognition and engages in direct ligand binding. Notably, a different rotameric state was assumed in the latter complexes, which allows Arg40 binding to the ligands with the longer side chain at the -3 position[6].

In addition to the conformational change, the intrinsic dynamics of Arg40 is different between the two crystal forms, and is significantly perturbed by the crystal packing. In *P*3_1_21-PDZ1, the mean anisotropy of Arg40 atoms is 0.337 (σ = 0.070), which is considerably more anisotropic than that in *P*2_1_-PDZ1 (*A* = 0.489, σ = 0.133) (Table 2). The majority of the largest anisotropic differences are located in the main chain region, which appear to correspond to the different crystal packing environments (Figure 3C). In *P*3_1_21-PDZ1, the thermal ellipsoids for the main chain atoms of Arg40 are prolate, with the longest principal axis oriented roughly parallel with the side chain direction, indicating that displacements of the Arg40 backbone are least constrained along the side chain and most constrained in directions orthogonal to the side chain. This result contrasts sharply to the more isotropic displacements in *P*2_1_-PDZ1, consistent with the extensive crystal packing and the fact that the orientations of the principal axes of the side chain fluctuations correspond closely to those of nearby neighboring atoms. Together, our crystallographic analysis demonstrates that residue Arg40 is intrinsically flexible, capable of exploring large conformational space, or visiting different conformations required for binding multiple partners.

**Table 2:** Isotropic B-factor and anisotropy.

Residue	Arg40		His27		His29		Glu43		Ser-3∶1^c^		Ser-3∶2	Protein (aa 9–94)		Peptide (aa 95–99)	
Space group	*P*2_1_	*P*3_1_21	*P*2_1_	*P*3_1_21	*P*2_1_	*P*3_1_21	*P*2_1_	*P*3_1_21	*P*2_1_	*P*3_1_21	*P*2_1_	*P*2_1_	*P*3_1_21	*P*2_1_	*P*3_1_21
**Isotropic B-factor (Å^2^)**															
All atoms	9.2	12.9	6.0	13.5	14.4	13.1	11.6	13.8	9.2	12.0	8.4	14.8	17.2	9.7	13.7
	(1.8)^a^	(1.5)	(0.6)	(2.5)	(2.4)	(1.7)	(1.5)	(3.6)	(0.7)	(1.2)	(0.6)				
Main-chain	5.8	9.5	5.1	9.3	11.0	9.9	10.4	10.2	8.5	11.8	8.4	13.7	15.3	8.3	12.1
	(0.0)	(0.1)	(0.0)	(0.0)	(0.2)	(0.1)	(0.1)	(0.2)	(0.1)	(0.1)	(0.1)	(1.0)	(1.3)	(0.7)	(1.3)
Side-chain	11.2	14.9	6.6	16.4	16.7	15.2	12.5	16.7	10.6	12.2	8.6	16.0	19.4	11.3	15.6
	(0.2)	(0.1)	(0.1)	(0.2)	(0.1)	(0.1)	(0.1)	(0.3)	(0.1)	(0.1)	(0.0)	(1.4)	(2.6)	**(1.1)**	(1.6)
**Anisotropy** b															
All atoms	0.489	0.337	0.608	0.330	0.484	0.470	0.319	0.352	0.447	0.358	0.349	0.430	0.424	0.482	0.432
	(0.133)	(0.070)	(0.088)	(0.054)	(0.174)	(0.086)	(0.062)	(0.061)	(0.118)	(0.082)	(0.066)	(0.136)	(0.118)	(0.119)	(0.126)
Main chain	0.585	0.309	0.681	0.357	0.296	0.386	0.276	0.332	0.376	0.303	0.324	0.411	0.411	0.436	0.386
	(0.084)	(0.017)	(0.071)	(0.023)	(0.046)	(0.059)	(0.043)	(0.030)	(0.069)	(0.034)	(0.066)	(0.141)	(0.100)	(0.132)	(0.118)
Side chain	0.435	0.353	0.560	0.312	0.610	0.526	0.354	0.368	0.588	0.467	0.398	0.450	0.439	0.536	0.491
	(0.125)	(0.083)	(0.062)	(0.061)	(0.097)	(0.046)	(0.053)	(0.073)	(0.045)	(0.001)	(0.022)	(0.128)	(0.134)	(0.074)	(0.115)

a Numbers in parentheses refer to standard deviation.

b The mean anisotropy *A* is defined as the ratio of the smallest to the largest eigenvalue of *U*, where *U* is ADP tensor.

c Ser-3∶1 conformation 1; Ser-3∶2 conformation 2.

**Figure 4:**
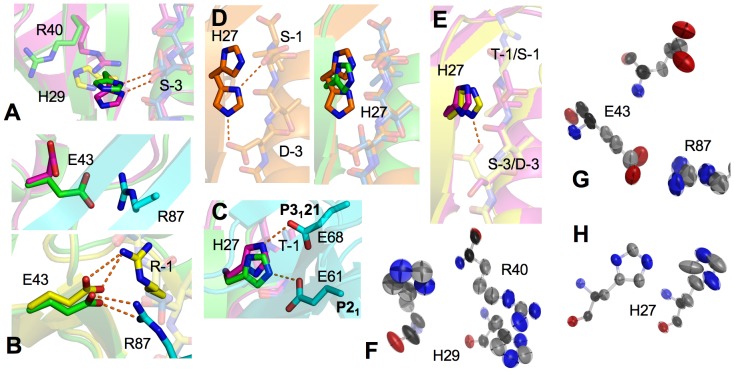
Conformational differences of His29, Glu43, and His27 between two crystal forms. (A) Superposition of His29 of *P*2_1_-PDZ1 (green), *P*3_1_21-PDZ1 (magenta), and PDZ1-CFTR (yellow). (B) Comparative view of Glu43 of *P*2_1_-PDZ1 and *P*3_1_21-PDZ1 shown together with *P*2_1_-PDZ1 symmetry-related molecules (top); superposition of Glu43 of *P*2_1_-PDZ1 and PDZ1-CFTR (bottom). (C) Superposition of His27 of *P*2_1_-PDZ1 and *P*3_1_21-PDZ1. Hydrogen bonds are illustrated as orange broken lines, and residues from the symmetry-related molecules are colored in cyan. (D) Left: dual positioning of His27 of NHERF2 PDZ1 (PDB code: 2OCS) and right: superposition of His27 of *P*2_1_-PDZ1 (green) and NHERF2 PDZ1 (orange). (E) Superposition of His27 of *P*3_1_21-PDZ1 and PDZ1-CFTR. (F), (G), and (H) Thermal ellipsoid representation of His29, Glu43, and His27 of *P*2_1_-PDZ1 (top/left) and *P*3_1_21-PDZ1 (bottom/right).

Another large conformational difference occurs at His29, a residue that plays a key role in Ser-3 recognition[6]. This difference is not the direct result of crystal packing, as neither crystal form involves His29 in the lattice interface, and the distance from His29 to the nearest neighboring atom is 4.6 Å for *P*3_1_21-PDZ1 and 6.2 Å for *P*2_1_-PDZ1. However, through altering intramolecular interactions, the crystal packing has an indirect impact on His29 conformation. In particular, different His29 structures in different crystal forms are the result of altered local environments with altered Arg40 conformations (Figure 4A and S1). In *P*3_1_21-PDZ1, the imidazole ring of His29 is held down by the guanido group of Arg40 via parallel stacking interactions, whereas in *P*2_1_-PDZ1, the packing-induced reorientation of the Arg40 side chain leads to breakage of such contacts, allowing His29 to adopt a more relaxed, ∼25° upward-tilted rotamer. In both crystal forms, residue His29 maintains direct hydrogen bonding to the Ser-3 hydroxyl, but this is achieved with a concerted change in the peptide structure. Specifically, the movement of the His29 side chain induces a corresponding movement in the side chain of Ser-3, which preserves the His29/Ser-3 contacts and ligand specific recognition (Figure 4A). Note the interaction of His29 with the -3 residue is dependent on the types of ligands; when binding to different ligands, the side chain of His29 can adopt very different conformers[6]. For example, in the PDZ1-CFTR complex[13], the side chain of His29 is completely oriented away from the CFTR peptide, adopting a conformation that is unable to interact with the ligand (Figure 4A). A similar conformer has been observed in PDZ1-β2AR and PDZ1-PDGFR[25], where the -3 residue (Asp-3) of both complexes is common to the PDZ1-CFTR complex. In addition, the intrinsic dynamics of His29 is discernible from the anisotropic displacement parameters (ADPs) of the structures. The mean anisotropy of His29 in the two crystal forms are very similar, 0.484 (σ = 0.174) for *P*2_1_-PDZ1 and 0.470 (σ = 0.086) for *P*3_1_21-PDZ1. However, the anisotropy of the main chain atoms and the side chain atoms is inversely different (Table 2). In *P*2_1_-PDZ1, His29 exhibits the higher main chain anisotropy, the principle axes of which match excellently with the direction of the displacements deduced from the structural alignment (Figure 4F). This indicates that His29 has an intrinsic propensity to undergo this movement. The higher side chain anisotropy of His29 in *P*3_1_21-PDZ1 appears to be related to the Arg40/His29 interaction, as the similar ADP magnitudes and orientations are observed for the Arg40 guanidinium and the His29 imidazole ring. These findings indicate that similar to Arg40, His29 is also intrinsically flexible and contributes to ligand specific binding and recognition.

Intriguingly, the peptide recognition by His29 and Arg40 appears to be mutually exclusive, as they occupy the overlapping space when binding to specific ligands. For instance, in PDZ1-CFTR, the interaction between Arg40 and Asp-3 induced steric effects that prevented His29 from ligand binding[13], [25]. In PDZ1-CXCR2, binding of His29 to Ser-3 caused a “kink” in Arg40's side chain resulting in the effective blockage of the Arg40-CXCR2 interaction[6]. This mutually exclusive peptide recognition may be advantageous, as the combined effects of individual recognition may increase PDZ1 robustness of ligand binding, or its capacity to interact with multiple ligands. The blend of His29 and Arg40 chemical properties, including hydrogen bonding, aromaticity, charge, and their intrinsic flexibility, may allow PDZ1 recognition of different -3 side chains. Interestingly, peptide affinity selection experiments showed that PDZ1 has no apparent amino acid preference for -3 position of peptides, capable of binding the peptides with -3 side chains of different size and polarity[32]. These suggest PDZ1 promiscuity may be due to multiplicity of possible binding modes that use different structural elements for binding structurally different ligands. They also indicate that the functional interplay between different peptide recognition residues requires a flexible binding pocket and the ability of this pocket to adopt significantly different conformations.

The third notable conformational difference between the two crystal forms is at Glu43, which, together with His29 and Arg40, provides evidence that substantial structural flexibility is present in the PDZ1 peptide-binding pocket. Specifically, in *P*3_1_21-PDZ1, Glu43 is not engaged in any crystal contacts, having an upward-folded, solvent-pointing side-chain conformation (Figure 4B and S1). In contrast, in *P*2_1_-PDZ1, due to the contact with the Arg87 guanidinium of a neighboring molecule, the side chain of Glu43 adopts a distinct conformation that stretches out towards the bound ligand. This stretched conformation and its Arg-interacting ability, are reminiscent of PDZ1-CFTR interaction. Similar Glu43 conformation observed in the PDZ1-CFTR structure is required for specific binding with the Arg-1 of the ligand[13]. Intriguingly, comparing the structures of PDZ1-CFTR and *P*2_1_-PDZ1 reveals that the positions of Arg-1 and Arg87 are completely different from one another, and they show no spatial overlap, approaching Glu43 from opposite directions (Figure 4B). As a result, significant differences exist in the salt bridge interaction scheme between the two crystal forms. In PDZ1-CFTR, the Oε2 atom of Glu43 makes bifurcated hydrogen bonds with Nε and Nη2 of Arg-1, whereas in *P*2_1_-PDZ1, the carboxylate oxygens of Glu43 are involved in separate hydrogen bonding to Nη1 and Nη2 of Arg87. This difference indicates that the stretched Glu43 conformation adopted in *P*2_1_-PDZ1 may be robust in Arg recognition, capable of binding Arg with different orientations. This conclusion is consistent with affinity selection experiments that showed NHERF1 PDZ1 prefers ligands with Arg at the -1 position, and the affinity of the PDZ1-ligand interaction can be reduced by mutation of Arg to Ala, Phe, Leu, or Glu[13], [32]. Given the recognized importance of Glu43 in peptide recognition[13], together with its ability to adopt different conformations upon binding different ligands[6], the crystal packing-induced conformational change in Glu43 provides further evidence for its structural adaptability, consistent with general proposition that PDZ flexibility contributes to PDZ promiscuity. More evidence in favor of this interpretation is provided by the observation that the crystal packing has a significant impact on the Glu43 anisotropic displacement parameters. In both crystal forms Glu43 exhibits strong anisotropy (*P*2_1_: *A* = 0.319; *P*3_1_21: *A* = 0.352), but the nature and orientations of their ADPs are discernibly different. In *P*3_1_21-PDZ1, the ADP orientations of the Glu43 atoms are not harmonized, whereas in *P*2_1_-PDZ1, the principal axes of the uniformly oriented ellipsoids correlate with the direction of the neighboring Arg87 fluctuations, indicating the dynamic adaptation of Glu43 to different crystal packing environments (Figure 4G).

Finally, the flexible nature of the PDZ1 peptide-binding pocket is evident in the observation that His27, which packs against -1 residue of the ligand, has different conformations in different crystal forms. The differences include a tilt of the side chain by 12° along the ligand and a 180° flip of the imidazole ring around the Cβ-Cγ bond (Figure 4C and S2). As a result of this reorientation, the imidazole ring is 1.0 Å closer to Ser-3 in *P*2_1_-PDZ1 than in *P*3_1_21-PDZ1, and there is an overall of 2.3 Å displacement between its Nε2 atoms. Note that the flip of the His27 imidazole ring does not significantly affect the His27/Thr-1 interaction, as the plane of the imidazole ring in the two crystals is similarly oriented after flipping, and the σ-π stacking interaction between His27 and the Thr-1 hydroxyl is essentially independent of altered Nε2 positions. Nonetheless, the difference in Nε2 positioning is a manifestation of different crystal packing environments. In both crystal forms, the imidazole ring of His27 is involved in crystal contacts but interacts with different symmetry-related residues. In *P*3_1_21-PDZ1, the Nε2 atom of His27 makes a hydrogen bond with the side chain Oε1 of Glu68, whereas in *P*2_1_-PDZ1, it is hydrogen-bonded to the equivalent oxygen from Glu61. As shown in structure alignment, the side chain of Glu61 is similar in orientation to Glu68, but slides more than 5 Å along the peptide binding cleft (Figure 4C). Remarkably, the direction of this shift corresponds to the direction of the His27 conformational change, suggesting the intrinsic flexibility of His27 that has the ability to adapt to different environments. This conclusion is supported by the observed anisotropic displacement parameters of His27 that differ dramatically between the two crystal forms (*P*2_1_: *A* = 0.608; *P*3_1_21: *A* = 0.330), and by the observation that the principle axes of the His27 ADPs correspond to the direction of the predicted structural changes (Figure 4H). The intrinsic flexibility of His27 is also evident from prior findings that the His27 of NHERF2 PDZ1 shows dramatically double conformations; one conformer stacks with -1 residue and the other simultaneously interacts with both -1 and -3 residues of a ligand (Figure 4D). Because NHERF2 PDZ1 His27 corresponds to NHERF1 PDZ1 His27, this implies that the conserved His27 is capable of exploring a large conformational space for promiscuous binding of various peptide sequences. It is intriguing to note the conformations of His27 are different when NHERF1 PDZ1 binds to different ligands. In PDZ1-CFTR, -β2AR, and -PDGFR, the conformations of His27 are highly superimposable, making a direct hydrogen bond to the common -3 residue (Asp-3) and a ligand-indiscriminative contact with the Cβ atom of the -1 side chain (Figure 4E). In contrast, in PDZ1-CXCR2 (*P*3_1_21-PDZ1), the imidazole ring of His27 rotates 20° to accommodate the Thr-1 hydroxyl, and is positioned 0.5 Å further from -3 position of the ligand due to the lack of specific hydrogen binding with the shorter Ser-3 side chain. These differences reflect the relationship between His27 conformations and PDZ1 promiscuity as well as the importance of His27 flexibility in binding different ligands.

### Different Modes of CXCR2 Peptide Interaction

**Figure 5:**
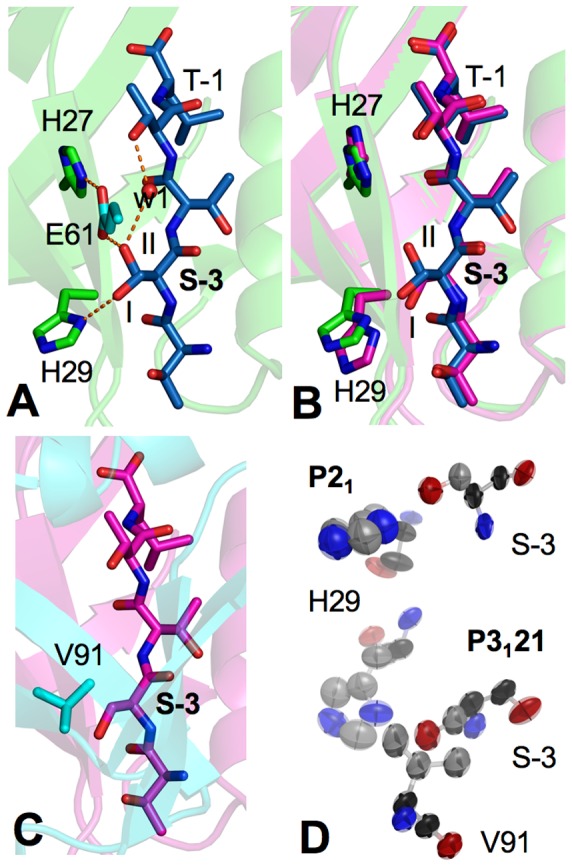
Distinct modes of CXCR2 peptide interaction. (A) Double conformation of Ser-3 in *P*2_1_-PDZ1. Residues of the CXCR2 peptide are represented by sticks with their carbon atoms colored in blue. PDZ1 residues are shown in green, while its symmetry-related residue is shown in cyan. Orange broken lines depict hydrogen bonds, and the labels I and II indicate individual conformers of Ser-3 double conformation. (B) Superposition of the CXCR2 peptides of *P*2_1_-PDZ1 (blue) and *P*3_1_21-PDZ1 (magenta). (C) Ser-3 crystal contacts in *P*3_1_21-PDZ1. (D) Thermal ellipsoid representation of Ser-3 interaction in *P*2_1_-PDZ1 (top) and *P*3_1_21-PDZ1 (bottom).

One notable difference between the peptides is at the Ser-3 side chain, which adopts a double conformation in *P*2_1_-PDZ1, but only one conformation in *P*3_1_21-PDZ1 (Figure 5). This difference indicates that similar to PDZ1, the bound ligand also exhibits significant flexibility, capable of assuming different conformations in different environments. Specifically, a 130° rotation around the Cα-Cβ bond relates the two Ser-3 conformations present in the *P*2_1_ crystal (Figure 5). One conformation is similar to the one observed for *P*3_1_21-PDZ1 (conformation 1), while the other represents a new conformer with the side chain pointing to the opposite direction of the ligand (conformation 2). In *P*2_1_-PDZ1, the two Ser-3 conformers are involved in completely different interaction networks resulting in two distinct modes of interaction with PDZ1. For conformation 1, the hydroxyl group of Ser-3 hydrogen bonds to the His29 imidazole ring, whereas in conformation 2, the Ser-3 side chain is stabilized by a van der Waals contact to the His27 Cδ2 atom and a hydrogen bond to a symmetry-related neighboring residue (Glu61) (Figure 5A and S1). Intriguingly, conformation 2 also engages in intrapeptide interaction and forms a water-mediated hydrogen bond with the Thr-1 side chain hydroxyl. It appears this dual positioning occurring in *P*2_1_-PDZ1 but not in *P*3_1_21-PDZ1 is due to the dramatic differences in crystal packing between the two crystal forms. In *P*2_1_-PDZ1, conformation 1 is not involved in any crystal contacts, whereas in *P*3_1_21-PDZ1, the tight packing between Ser-3 and Val91 may restrict the Ser-3 conformational flexibility and impedes the possible rotation of its side chain (Figure 5C). This interpretation is supported by the fact that the axes of Ser-3 and Val91 fluctuations remarkably match with each other in *P*3_1_21-PDZ1, whereas the lack of the crystal contact in *P*2_1_-PDZ1 results in apparently coupled motion between Ser-3 and His29, which is not found in *P*3_1_21-PDZ1 (Figure 5D). Together, these observations provide some evidence that the intrinsic dynamics of the peptide ligand allows for interactions with different peptide recognition residues. While the functional significance of this dynamical response is unknown, future studies should be directed toward evaluation of its effects on PDZ specificity and promiscuity; especially to determine whether the peptide flexibility is important for single peptides to bind to distinct PDZ domains. It is of particular interest to note the recognition of a peptide-loaded MHC molecule (major histocompatibility complex) by the cognate T-cell receptor depends on the dynamics properties of the peptides, and differential peptide flexibility resulting from MHC polymorphisms can broaden and expand T-cell receptor reactivity[33], [34].

## Discussion

That different PDZ1 conformations are captured by different crystal forms is not surprising in itself, but to the extent it suggests the conformational space available to certain regions of the protein. The set of different crystal structures is thus particularly informative since it may represent different PDZ1 conformational states and reflects the protein's functional dynamics. One can argue that none of the PDZ1 crystal structures correspond exactly to native substates because of the influence of crystal packing artifacts. Although this concern is likely to be somewhat valid, analysis of thermal factors of nonisomorphous lysozyme structures suggests that the crystal lattice does not just force some random conformational changes onto the molecule, but rather the molecule moves along essential eigenvectors to adapt to different lattice environments[35], [36]. Furthermore, the agreement of the residues undergoing the packing-induced conformational change with the residues involved in allosteric transition in response to ligand binding is in support of use of crystal forms for consolidating PDZ1 structural data, or gaining insights into potential ligand binding mechanisms (Figure 3). Thus the present *P*2_1_ crystal form plus the four original liganded structures in the *P*3_1_21 space group provide in total five independent views of PDZ1 bound to its targets. Although these structures likely account for only a tiny portion of the entire conformational space, they allow us to at least tentatively begin to sketch the mechanism that describes how the protein works, and provide the basis for consideration of the PDZ1 structural dynamics and the mechanism by which PDZ1 flexibility contributes to PDZ1 promiscuity. On the other hand, given the exceptional importance of NHERF1 in tumorigenesis and inflammation[2], [3], [7], the knowledge of individual PDZ1 conformational states may be valuable in developing new methods and strategies for selective drug design. For instance, this information can be used to describe binding site flexibility that may allow for accurate modeling of PDZ1-inhibitor interactions. The information also allows for the use of ensemble docking in compound screening, and may contribute to druggable hot-spot identification, and the designing of highly selective compounds[37], [38]. Taken together, the collection of available PDZ1 structures provides insight into the PDZ1 conformational dynamics and the structural explanations of how PDZ1 is able to bind to different ligands. It is no doubt that further understanding of the rules that underlying the ligand-binding site dynamics will benefit from continued studies of PDZ1 liganded structures in different crystal forms.

## Materials and Methods

### Protein Expression and Purification

A DNA fragment encoding the human NHERF1 PDZ1 (residues 11–94), and having the C-terminal extension TSTTL that corresponds to residues 356–360 of human CXCR2, was amplified using PCR and cloned in the pSUMO vector[6]. The resulting clone containing a N-terminal His6-SUMO tag was transformed into *Escherichia coli* BL21 Condon Plus (DE3) cells for protein expression. The transformants were grown to an OD600 (optical density at 600 nm) of 0.4 at 37°C in LB medium, and then induced with 0.1 mM isopropylthio-β-D-galactoside and grown an additional 16 hours at 15°C. The cells were harvested by centrifugation and lysed by French Press. The soluble fraction was then subjected to Ni^2+^ affinity chromatography purification, followed by the cleavage of the His6-SUMO tag with yeast SUMO Protease 1. PDZ1 was separated from the cleaved tag by a second Ni^2+^ affinity chromatography and further purified by size-exclusion chromatography. Finally, the protein was concentrated to 40–50 mg/ml in a buffer containing 20 mM Tris–HCl (pH 8.0), 150 mM NaCl, 1 mM β-mercaptoethanol (BME), and 5% glycerol.

### Crystallization, Data Collection and Structure Determination

Crystals were grown by the hanging-drop vapor-diffusion method by mixing the protein (∼25 mg/ml) with an equal volume of reservoir solution containing 100 mM sodium acetate, pH 4.8, 0.2 M ammonium acetate, 25% PEG4000 at 20°C. Crystals typically appeared overnight and continued to grow to full size in 3–4 days. Before X-ray diffraction data collection, crystals were cryoprotected in a solution containing mother liquor and 25% glycerol and flash cooled in liquid nitrogen. The data were collected at 100 K at beamline 21-ID-F at the Advanced Photon Source (Argonne, IL) and processed and scaled using the program HKL2000[39]. Crystals belong to space group *P*2_1_ with unit cell dimensions a = 26.6 Å, b = 45.5 Å, c = 33.4 Å, β = 109.7°, and one molecule in the asymmetric unit. The structure was solved by the molecular replacement method with program PHASER[40] using the *P*3_1_21-PDZ1 structure (PDB code: 4JL7) as a search model. The structure modeling was carried out in COOT[41], and refinement was performed with PHENIX[42]. The riding hydrogen and ADP features were included in the refinement, and no ADP restraint was employed. The final models were analyzed and validated with Molprobity[29]. The ADPs were analyzed using ANISOANL[30] and the PARVATI server[43]. All figures of 3D representations of the *P*2_1_-PDZ1 structure were made with PyMOL (www.pymol.org).

### Protein Data Bank Accession Number

Coordinates and structure factors have been deposited in the Protein Data Bank with accession number 4MPA (*P*2_1_-PDZ1) and 4N6X (*P*3_1_21-PDZ1).

## Supporting Information

Figure S1 **Electron density of selected residues.** The left panel, *P*2_1_-PDZ1; the right panel, *P*3_1_21-PDZ1. Residues are depicted by sticks overlaid with 2F_o_−F_c_ omit map calculated at 1.1 Å for *P*2_1_-PDZ1 and 1.16 Å for *P*3_1_21-PDZ1, and contoured at 1.5 σ.
(TIF)

Figure S2 **Electron density of His27 at high contour level.** The left panel, *P*2_1_-PDZ1; the right panel, *P*3_1_21-PDZ1. His27 is depicted by sticks overlaid with 2F_o_−F_c_ omit map calculated at 1.1 Å for *P*2_1_-PDZ1 and 1.16 Å for *P*3_1_21-PDZ1. The maps are contoured at 5.0 σ, which reveal the densities at the position of nitrogen atoms are stronger than the densities at the position of carbon atoms (Nε2 vs. Cε1; Nσ1 vs. Cσ2). The B factors of the side chain atoms are shown in parentheses after the atom names.
(TIF)
