# Supplementary figures and images for: New Conformational State of NHERF1-CXCR2 Signaling Complex Captured by Crystal Lattice Trapping
Source: PLoS One. 2013 Dec 10;8(12):e81904. doi: 10.1371/journal.pone.0081904 (PMC3858284; doi:10.1371/journal.pone.0081904)

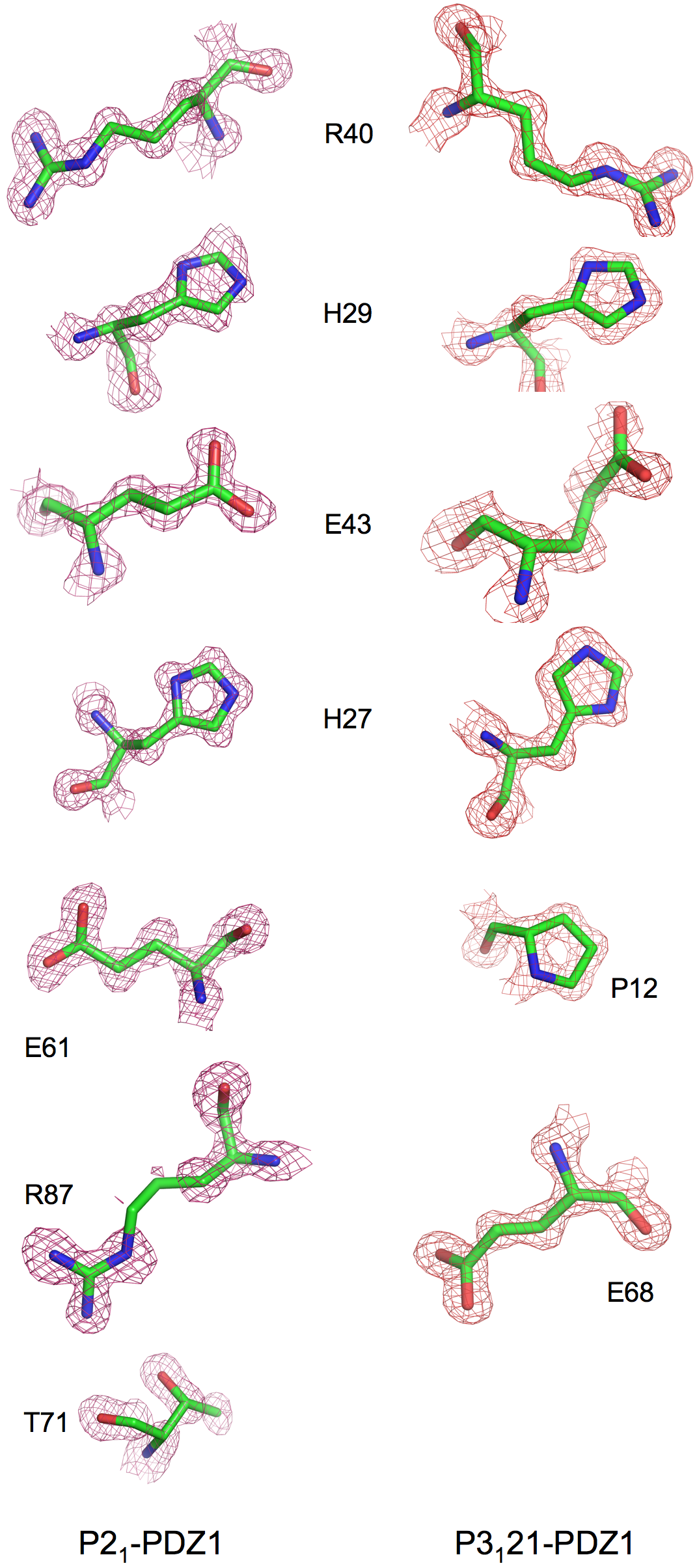

Supplement: Figure S1 — Electron density of selected residues. The left panel, P21-PDZ1; the right panel, P3121-PDZ1. Residues are depicted by sticks overlaid with 2Fo−Fc omit map calculated at 1.1 Å for P21-PDZ1 and 1.16 Å for P3121-PDZ1, and contoured at 1.5 σ. (TIF) [file pone.0081904.s001.tif]

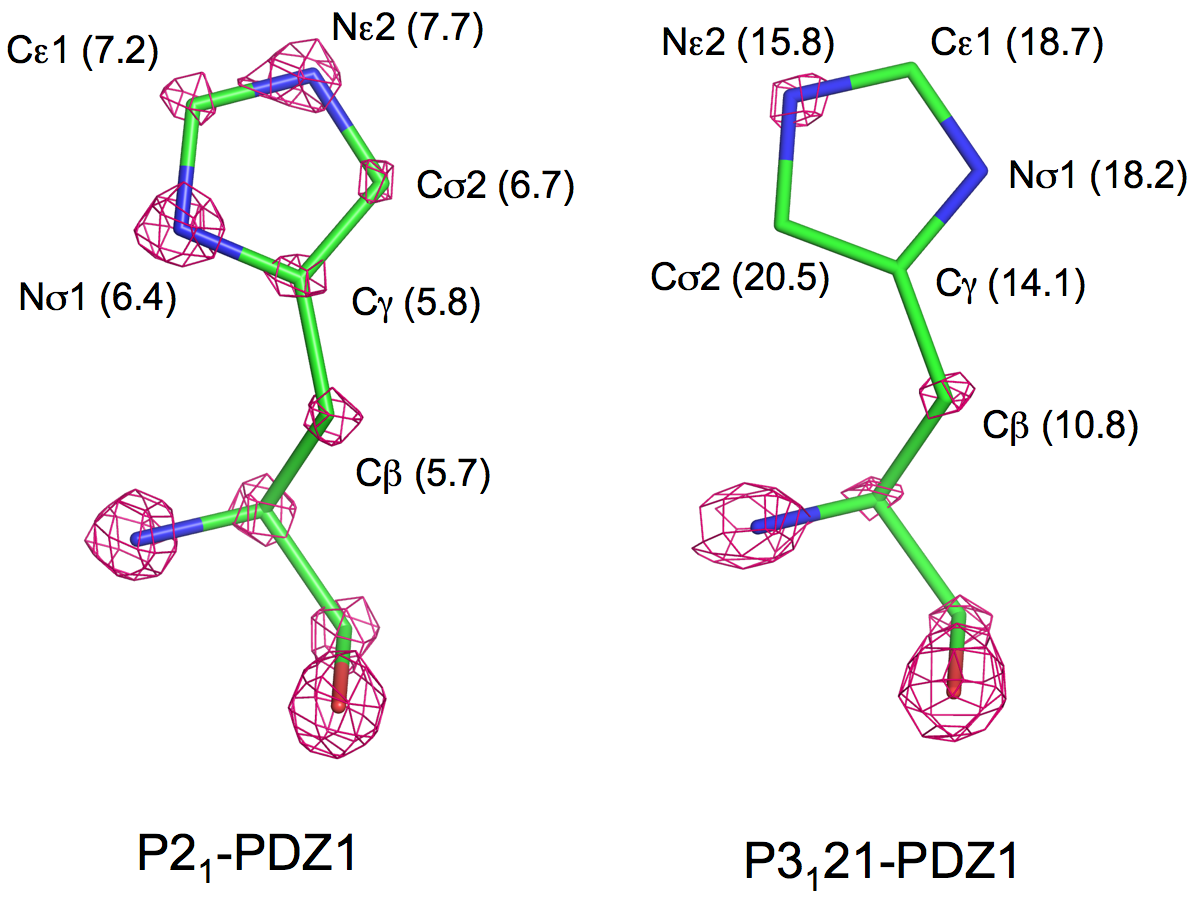

Supplement: Figure S2 — Electron density of His27 at high contour level. The left panel, P21-PDZ1; the right panel, P3121-PDZ1. His27 is depicted by sticks overlaid with 2Fo−Fc omit map calculated at 1.1 Å for P21-PDZ1 and 1.16 Å for P3121-PDZ1. The maps are contoured at 5.0 σ, which reveal the densities at the position of nitrogen atoms are stronger than the densities at the position of carbon atoms (Nε2 vs. Cε1; Nσ1 vs. Cσ2). The B factors of the side chain atoms are shown in parentheses after the atom names. (TIF) [file pone.0081904.s002.tif]
